# G6PD deficiency: imbalance of functional dichotomy contributing to the severity of COVID-19

**DOI:** 10.2217/fmb-2021-0299

**Published:** 2022-07-26

**Authors:** Abir Mondal, Soumyadeep Mukherjee, Waseem Dar, Prince Upadhyay, Anand Ranganathan, Soumya Pati, Shailja Singh

**Affiliations:** ^1^Department of Life Sciences, Neurobiology & Disease Modelling Laboratory, Host-Pathogen Interactions & Disease Modelling Group, School of Natural Sciences, Shiv Nadar University, Greater Noida, 201314, India; ^2^Special Centre for Molecular Medicine, Jawaharlal Nehru University, New Delhi, India

**Keywords:** COVID-19, G6PD deficiency, genetic factor, inflammation, oxidative stress

## Abstract

Human COVID-19 has affected more than 491 million people worldwide. It has caused over 6.1 million deaths and has especially perpetrated a high number of casualties among the elderly and those with comorbid illnesses. COVID-19 triggers a pro-oxidant response, leading to the production of reactive oxygen species (ROS) as a common innate defense mechanism. However, ROS are regulated by a key enzyme called G6PD via the production of reduced nicotinamide adenine dinucleotide phosphate (NADPH), which controls the generation and removal of ROS in a tissue-specific manner. Therefore, a deficiency of G6PD can lead to the dysregulation of ROS, which causes a severe inflammatory response in COVID-19 patients. This report highlights the G6PD dichotomy in the regulation of ROS and inflammatory responses, as well as its deficiency in severity among COVID-19 patients.

G6PD is a rate-limiting enzyme of the pentose phosphate pathway, and it mediates the production of 6-phosphogluconate and NADPH [1]; 6-phosphogluconate acts as a substrate for the generation of ribose sugar used for nucleotide biosynthesis. At the same time, NADPH plays a vital role in one-carbon metabolism and the regulation of cellular redox equilibrium [2]. Therefore, a deficiency of G6PD alters physiological functions and leads to the development of several disorders. G6PD deficiency is a common X-linked recessive enzyme deficiency disorder in humans, affecting more than 400 million people worldwide, with a high prevalence in persons of African, Asian and Mediterranean descent [3]. More than 400 *G6PD* variants have been found, and among them 165 variants mediate diverse pathophysiology [4]. Based on the degree of deficiency, WHO has classified G6PD deficiency into five classes [5]. G6PD deficiency often leads to the development of hemolytic anemia, hyperbilirubinemia, kernicterus and neurological and neurodevelopmental disorders [4,6–11]. The previous reports also suggested that G6PD deficiency increases the risk of cardiovascular disorder among populations in the USA, the Mediterranean region and China [12–14]. The function of G6PD in the regulation of redox equilibrium varies based on the cell type. For example, G6PD-derived NADPH promotes ROS production via NADPH oxidase in macrophages to neutralize foreign pathogens and initiates a proinflammatory response via the production of cytokines [15,16]. NADPH also helps neutralize ROS in a glutathione-dependent manner in other cell types such as red blood cells, neurons and lung alveolar cells [4,8,11]. G6PD-deficient cells are also characterized by a low reduced glutathione-to-oxidized glutathione ratio and the accumulation of lipid peroxidation products, which leads to cataractogenesis, glycation of hemoglobin and cellular damage *in vitro* [17,18]. The reduction in the amount of reduced glutathione levels is also associated with a deficiency of vitamin D and the inability to regulate the oxidative cellular environment during infection [19]. Overall, G6PD-deficient patients are more susceptible to fibrosis, autoimmune diseases, metabolic disorders and neurodegenerative disease. Therefore, G6PD deficiency and the NADPH–ROS axis orchestrate a multifaceted pathophysiological response in general, which becomes extremely fatal during COVID-19.

However, SARS-CoV-2 primarily affects the respiratory system, although other organs are also involved in the pathophysiology. Confirmed and reported cases of COVID-19 have a wide range of symptoms such as fever, cough, sore throat, loss of taste or smell, diarrhea and headache [20]. Some individuals, especially elderly patients with comorbid illness, develop difficulty breathing. Recent reports suggest that COVID-19 accelerates cellular ROS generation [21–25]. Therefore, COVID-19 patients with G6PD deficiency can experience severe outcomes. In particular, uncontrolled ROS generation due to a deficiency of G6PD in alveolar lung cells leads to the destruction of lung alveoli, ultimately resulting in irreversible lung damage. Additionally, G6PD deficiency impedes innate immune response mediated by macrophages. Therefore, the balance between antioxidant and pro-oxidant pathways mediated by G6PD plays a fundamental role in regulating physiological function during COVID-19. This review discusses the importance of the functional dichotomy of G6PD in the management of COVID-19 infection. Further, it highlights that a deficiency of *G6PD* can be one of the genetic comorbid factors in the severity of COVID-19.

## Pathogenesis caused by COVID-19 infection & accelerated ROS production

**Figure 1.**
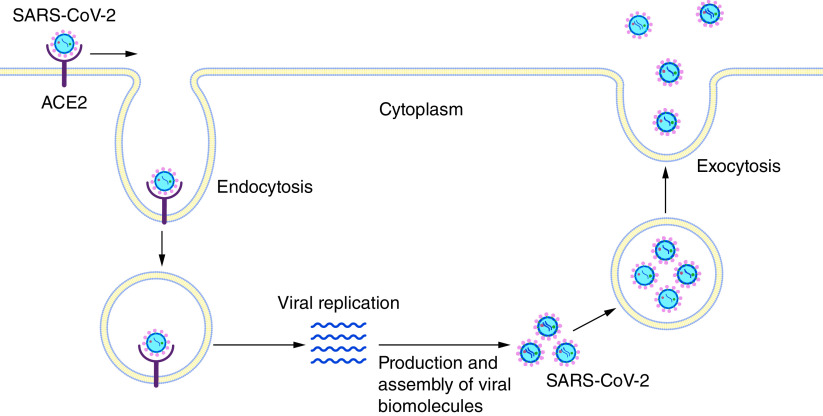
SARS-CoV-2 entry into and multiplication in host cells. SARS-CoV-2 enters host cells via receptor-mediated endocytosis after binding with the ACE2 receptor. Then, SARS-CoV-2 hijacks the host-cell replication machinery for proliferation and produces a large number of viruses. Later, those newly generated viruses are incorporated into membrane-bound vesicles for release by exocytosis.

SARS-CoV-2 also binds to ACE2 receptors to enter host cells [26–29]. Later, the viral genome replicates inside the host cells and produces many viral particles, which are released from the cells via exocytosis (Figure 1). Thus, SARS-CoV-2 activates innate immune responses to attenuate the growth of the virus. Neutrophils and macrophages are the key players of the innate immune response associated with COVID-19 disease [30,31]. These cells can release several proinflammatory cytokines, such as interleukins and TNF-α. Additionally, activated neutrophils release chromatin to trap and kill invading SARS-CoV-2 [32]. The chromatin trap is known as the neutrophil extracellular trap. Clinical studies have highlighted elevated neutrophil extracellular trap formation in COVID-19 patients [33,34].

**Figure 2.**
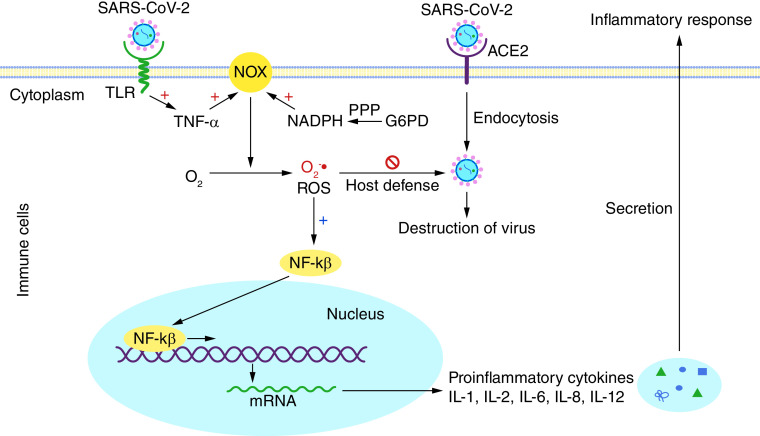
Oxidative stress and inflammation. Binding of SARS-CoV-2 with Toll-like receptors can lead to activation of TNF-α. Activated TNF-α facilitates reactive oxygen species production by NOX, and G6PD-derived NADPH directly helps in this process. The production of reactive oxygen species by immune cells is further utilized to kill viruses and generate proinflammatory cytokines in an NF-κβ-dependent manner. Later, cytokines are released from immune cells, causing an inflammatory response.

However, the innate immune response to COVID-19 is also mediated by Toll-like receptors present on the macrophages after binding with SARS-CoV-2 (Figure 2) [21,35]. Activation of Toll-like receptors promotes TNF-α-mediated inflammatory responses and ROS production for neutralizing SARS-CoV-2 by macrophages (Figure 2) [21,36]. However, excessive ROS also activate NRF2 pathways for maintaining redox equilibrium via the expression of *SOD*, *GST*, *catalase*, *HO-1* and *NQO1* etc [37–39]. Unfortunately, the inhibition of NRF2 pathways has been reported in COVID-19 patients [25,38,39]. Thus, accelerated ROS production leads to a severe inflammatory response, which ultimately destroys host cells. Additionally, SARS-CoV-2-mediated ROS generation promotes the production and secretion of proinflammatory cytokines in macrophages in an NF-κβ-dependent manner [32,40]. The release of cytokines triggers a massive immune response, which often destroys lung epithelium. The cytokine storm and acute respiratory distress due to the destruction of alveoli cause mortality in COVID-19 patients [41–46]. The mechanisms underlying lung dysfunction may depend on the degree of oxidative stress and innate immune activity. In addition, oxidative stress, inflammation, loss of alveolar cells and an excessive accumulation of extracellular matrix lead to lung fibrosis progress [47,48].

## Oxidative stress, inflammation & the functional dichotomy of G6PD

**Figure 3.**
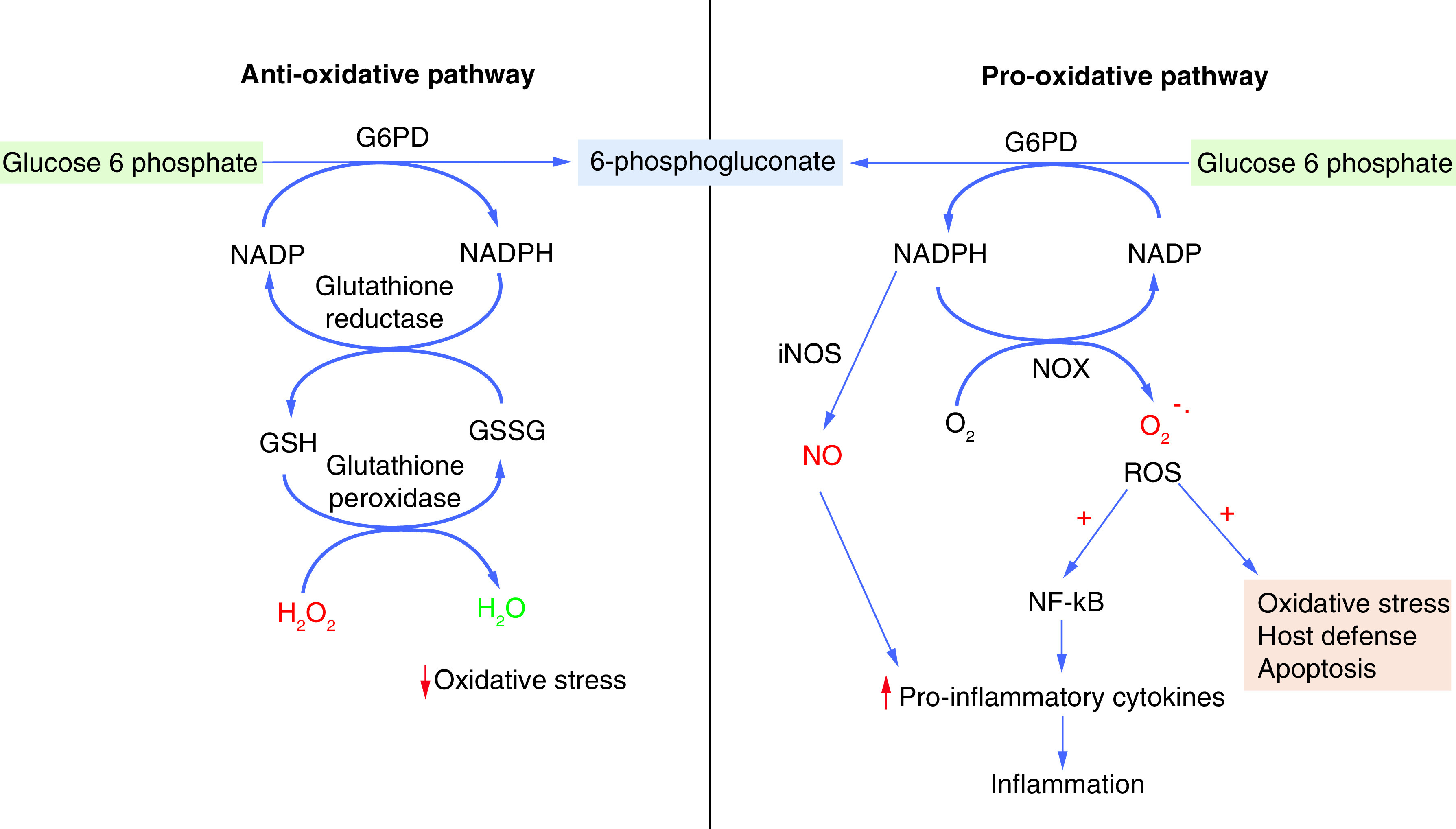
The functional dichotomy of G6PD. G6PD shows both pro-oxidative and antioxidative functions, depending on the cell type. The enzymatic activity of G6PD helps in the production of 6-phosphogluconate and NADPH. The NADPH can help in either the reduction of reactive oxygen species in a glutathione-dependent manner or the generation of reactive oxygen species via iNOS and NOX-dependent manner.

Superoxide free radicals are constantly generated inside every cell by enzymatic and nonenzymatic reactions in response to diverse stimuli. Enzymatic reactive oxygen species (ROS) production is associated with biochemical reactions catalyzed by NOX, XO and NOS. Besides, the mitochondria electron transport chain is the primary nonenzymatic source of cellular ROS generation [49,50]. However, ROS at the physiological concentrations can also function as second messengers and activate multiple signal transduction pathways within the cell, facilitating cellular growth, cytokine production and calcium signaling [51,52]. ROS generation by NOX in phagocytic cells such as macrophages and neutrophils is essential for the killing of foreign pathogens (Figure 2) [53]. On the other hand, ROS can cause direct injury to proteins, lipids and nucleic acids, leading to cell death. In general, several enzymes, such as G6PD, SOD, catalase, HQ-1 and NQO-1, regulate cellular redox equilibrium. However, decreased biosynthesis of redox regulators such as HO-1, SOD, catalase and NQO-1 is observed in COVID-19 patients due to the inhibition of NRF2 pathways [38,39]. Hence, G6PD plays a significant role in the regulation of ROS via the production of NADPH during COVID-19. However, G6PD mediates both pro-oxidative and antioxidative roles in the regulation of redox equilibrium, depending on the cell type (Figure 3). In relation to pro-oxidative function of G6PD, several reports claim that G6PD-derived NADPH is the primary substrate for ROS production by NOX and generates superoxide and nitric oxide [53–55]. Under stressful conditions, G6PD facilitates ROS generation in several cell types, including macrophages, granulocytes, adipocytes and myocardial cells [56]. For example, upregulation of G6PD and subsequent ROS production were observed in macrophages after lipopolysaccharide treatment [16]. Besides, macrophages utilize G6PD-derived NADPH to produce ROS that activate NF-κβ signaling [32]. In addition, NF-κβ induces the expression and secretion of proinflammatory cytokines such as IL-1, IL-2, IL-6, IL-8 and IL-12 (Figures 2 & 3) [21,32]. Suppression of G6PD activity downregulates NADPH oxidase, INOS and ROS production, which further mitigates the proinflammatory response in activated macrophages [57].

Red blood cells mainly depend on G6PD-derived NADPH for maintaining redox equilibrium compared with other cells. Therefore, a deficiency of G6PD causes oxidative stress, and subsequent hemolysis leads to hemolytic anemia [58–60]. Similarly, the role of G6PD deficiency has been linked with oxidative stress and neuroinflammation in neurodegenerative and neurodevelopmental disorders [11]. Generally, neurons are innately vulnerable to oxidative stress, and a reduced NADPH level due to G6PD deficiency causes neuroinflammation and loss of neurons [61]. A similar phenotype is observed in alveolar cells, resulting in the destruction of lung alveolar cells under oxidative stress caused by foreign pathogens [23,62–66].

## Inflammatory response due to ROS accumulation in G6PD-deficient COVID-19 patients

**Figure 4.**
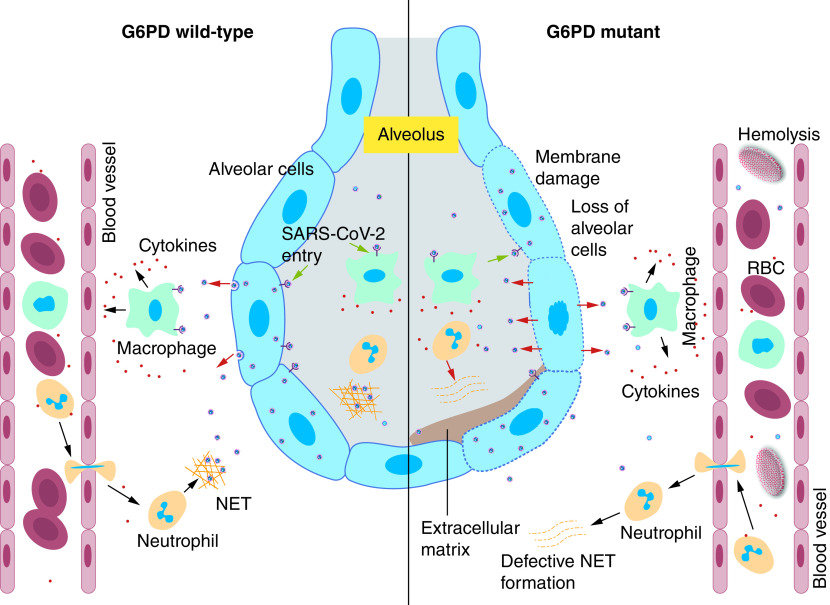
Oxidative stress, inflammation and the severity of COVID-19. SARS-CoV-2 enters and multiplies in alveolar cells. As a result, lung alveolar macrophages and neutrophils are activated and cause a cytokine storm. The activated neutrophils also release neutrophil extracellular traps for killing SARS-CoV-2. Therefore, a tightly regulated redox balance kills SARS-CoV-2 and aids recovery from COVID-19 in G6PD wild-type patients. On the contrary, defective neutrophil extracellular trap formation and unregulated reactive oxygen species generation fail to manage SARS-CoV-2 infection in G6PD-deficient patients. Further, uncontrolled reactive oxygen species also damage alveolar cells and facilitate the progression of lung fibrosis. Additionally, oxidative stress can trigger hemolysis in G6PD-deficient COVID-19 patients.

Macrophage- and neutrophil-mediated inflammatory response pathways get altered in G6PD-deficient patients, because G6PD-derived NADPH acts as a substrate for NOX and stimulates neutrophil extracellular trap formation by neutrophils (Figure 4) [32,67]. Therefore, G6PD deficiency leads to defective neutrophil extracellular trap formation (Figure 4) and NOX activity in the neutrophils of individuals affected by foreign pathogens [34]. Additionally, one of the byproducts of neutrophil extracellular trap is elastase, which causes hypertension, thrombosis and vasculitis in COVID-19 patients [68–70]. Further, neutrophils and macrophages are the main ROS producers during SARS-CoV-2 infection. Although ROS production by immune cells is required for the killing of COVID-19, but its activity could be neutralized by the enzymatic reactions of G6PD. However, in G6PD-deficient patients, the regulation of ROS is impeded. ROS accumulation inside cells can damage biomolecules such as proteins, lipids and nucleic acids, which ultimately causes cell death [71]. Uncontrolled ROS production damages lung alveolar cells and increases mucus secretion, which eventually causes difficulty breathing in COVID-19 patients (Figure 4) [23,62–65]. A recent report also suggests that G6PD deficiency activates monocytes and alters macrophage polarization, which functionally resembles the proinflammatory phenotype [15]. Additionally, oxidative stress in subcellular compartments can arrest proliferation and differentiation [72]. Therefore, a deficiency of G6PD acts as double-edged sword; macrophage function is compromised and excessive ROS accumulation destroys host cells during COVID-19 infection. Excessive ROS not only damages alveolar cells but also causes hemolysis (Figure 4). The loss of red blood cells may contribute to hypoxic respiratory failure in some patients with COVID-19 [73–75]. Elevated heme and hemoglobin levels in blood can further aggravate oxidative stress [73]. Additionally, several diagnostic markers are altered in G6PD-deficient COVID-19 patients compared with G6PD wild-type COVID-19 patients (Table 1). Therefore, G6PD deficiency, high ROS generation and the cytokine storm contribute to disease severity in COVID-19 patients, and it should be considered for future therapeutic development.

**Table 1.** Altered physiological parameters in COVID-19 patients along with G6PD deficiency.

Parameters	Level of marker in G6PD-deficient COVID-19 patients compared with G6PD wild-type COVID-19 patients	Ref.
C-reactive protein	High	[87]
D-dimer	High	[87]
Bilirubin in blood	High	[82,87]
Creatine	Low	[87]
Hemoglobin	Low	[83,85,87]
Acute respiratory distress syndrome	Present	[83 87]
Hemolysis and methemoglobinemia after hydroxychloroquine treatment	Increased	[82,85]
Ventilation support	Prolonged ventilation support	[87]

## Management of COVID-19 severity in G6PD-deficient patients

The SARS-CoV-2 genome is highly susceptible to mutation. Gain-of-function mutation in the SARS-CoV-2 genome is associated with a high rate of transmission, morbidity and ineffectiveness of developed vaccines worldwide. Hence, antiviral strategies that inhibit SARS-CoV-2 are urgently required. Apparently, oxidative stress during COVID-19 infection plays a multifaceted role in the severity of COVID-19. In addition, G6PD deficiency can pose a challenge to surviving COVID-19. One study found that α-lipoic acid can mitigate the vulnerability of G6PD deficiency *ex vivo* [76]. Thus, α-lipoic acid has been proposed as a treatment option for COVID-19 [77,78]. Hydroxychloroquine has also been proposed as a treatment for COVID-19. However, the use of hydroxychloroquine in G6PD-deficient patients may cause severe hemolysis, as the drug has oxidative properties [60,79,80]. The oxidative stress can be reduced by small-molecule activator of NRF2 pathway which could be a strategy to prevent the severity of COVID-19. A recent study identified that the NRF2 agonists 4-octyl-itaconate (4-OI) and clinically approved dimethyl fumarate stimulate a cellular antiviral pathway that effectively impedes the replication of SARS-CoV-2 *in vitro* [39]. In addition, 4-OI and dimethyl fumarate restrict host inflammatory responses during SARS-CoV-2 infection [39]. Though, the use of antioxidant therapy could be beneficial for COVID-19 patients but the extensive studies are required for validation [81,82].

## Conclusion

The invasion of the body by foreign pathogens causes the activation of immune responses tightly regulated by immune modulators. Alteration in the proper physiological function of immune mediators can cause a severe outcome. Excessive reactive oxygen species generation during COVID-19 infection can be regulated by cellular redox regulators, which further support recovery from infection. However, a deficiency of redox regulators, mainly G6PD, may cause severe outcomes, as discussed here. However, the degree of G6PD deficiency is a significant concern, as it may show diverse pathophysiology of COVID-19. Additionally, G6PD deficiency is a common X-linked recessive enzymopathy that most often affects men [83]. Similarly, recent studies showed higher mortality in male COVID-19 patients due to severe pneumonia and acute respiratory distress syndrome, indicating possible X-linked pathology [84]. Besides, preclinical studies using coronavirus-infected G6PD-deficient cells showed impaired cellular responses and aggravated oxidative damage [84]. Subsequently, COVID-19 studies on hospitalized patients with G6PD deficiency have shown that they require more oxygen supplementation and more prolonged mechanical ventilation support, indicating an increased severity of pneumonia, than G6PD wild-type patients [84]. Therefore, based on various reports, we can suggest that G6PD deficiency is one of the genetic factors contributing to the severity of COVID-19, and clinicians should consider that fact in the treatment of patients.

## Future perspective

Based on previous reports, we can speculate that every foreign pathogen can trigger severe pathological manifestations in patients carrying *G6PD* mutation. Notably, G6PD deficiency linked to diabetes and cardiovascular disorders might be one of the causative factors underlying COVID-19-associated comorbidities. Considering many variations in the *G6PD* gene and their clinical relevance, future research might focus on the development of mutation-specific small-molecule drugs and their validation *in vitro* and *in vivo*. AG-1, a newly discovered activator of G6PD that increases the enzymatic activity of the wild-type and mutant G6PD molecules [85], has opened a new avenue for designing novel small-molecule mimetics targeting G6PD-linked diseases. In addition to existing antioxidant therapeutics against COVID-19, such as vitamin D and reduced glutathione precursor L-cysteine may also reduce the severity of infection in patients with G6PD deficiency [19,86,87]. Further, repurposing the existing antioxidant drugs, such as N-acetyl cysteine, could be an alternative strategy for improving disease pathology.

**Executive summary**

**Pathogenesis caused by COVID-19**

- SARS-CoV-2 activates innate immune responses, which attenuate the growth of the virus.
- Activation of Toll-like receptors promotes TNF-α-mediated inflammatory responses and reactive oxygen species production for neutralizing SARS-CoV-2 by macrophages and other immune cells.
- Cytokine storm triggers a massive immune response, which often destroys lung epithelium.

**Functional dichotomy of G6PD**

- G6PD-derived NADPH can promote both pro-oxidative and antioxidative functions in a cell type-dependent manner.
- The pro-oxidative function of G6PD helps in the destruction of foreign pathogens by immune cells.
- The antioxidative function of G6PD scavenges free radicals and maintains cellular physiology.
- The functional dichotomy of G6PD plays an important role in regulating redox equilibrium and neutralizing foreign pathogens.

**Inflammation in G6PD-deficient COVID-19 patients**

- G6PD deficiency causes uncontrolled reactive oxygen species production, which damages lung alveolar cells and increases mucus secretion, eventually causing difficulty breathing in COVID-19 patients.
- Activation of NF-κβ signaling promotes a massive inflammatory response, which often destroys lung epithelium.
- G6PD deficiency leads to defective neutrophil extracellular trap formation, and a byproduct causes hypertension, thrombosis and vasculitis in COVID-19 patients.

**Management of COVID-19 severity in G6PD-deficient patients**

- Hydroxychloroquine has oxidative properties, and its use may lead to fatal consequences in COVID-19.
- NRF2 pathways can prevent the severity of COVID-19 via the reduction of oxidative stress.
- The use of NRF2 agonists such as 4-OI can prevent the severity of COVID-19 and other infectious diseases.

**G6PD deficiency as a genetic factor in COVID-19 severity**

- COVID-19 has shown higher mortality in male patients, indicating possible X-linked pathology, which is perfectly aligned with X-linked G6PD-deficiency disorder.
- Notably, G6PD deficiency is one of the genetic factors contributing to the severity of COVID-19.

**Future perspective**

- Research focusing on the development of small-molecule activators for G6PD variants should be a priority for the prevention of the severity of infectious diseases.
